# AI-Assisted Detection of Supraspinatus Tendon Pathologies Using a Hierarchical Deep Learning Model to Improve Clinical Applicability: Development and Evaluation Study

**DOI:** 10.2196/84804

**Published:** 2026-07-08

**Authors:** Kun-Hui Chen, Jacky Chung-Hao Wu, Hsin-Yu Chang, En-Rung Chiang, Hsuan-Hsiao Ma, Hsin-Yi Wang, Henry Horng-Shing Lu, Chih-Yu Yang

**Affiliations:** 1 Institute of Clinical Medicine, National Yang Ming Chiao Tung University, No. 155, Sec. 2, Linong Street, Taipei, Taiwan, 886 2- 2871-2121 ext 23103; 2 Department of Orthopaedics and Traumatology, Taipei Veterans General Hospital, Taipei, Taiwan; 3 Department of Surgery, School of Medicine, National Yang Ming Chiao Tung University, Taipei, Taiwan; 4 Center for Fundamental Science, Kaohsiung Medical University, Kaohsiung, Taiwan; 5 Biomedical Artificial Intelligence Academy, Kaohsiung Medical University, Kaohsiung, Taiwan; 6 Institute of Statistics, National Yang Ming Chiao Tung University, Hsinchu, Taiwan; 7 Department of Anaesthesiology, Taipei Veterans General Hospital, Taipei, Taiwan; 8 Department of Medical Research, Kaohsiung Medical University Hospital, Kaohsiung, Taiwan; 9 Department of Statistics and Data Science, Cornell University, Ithaca, NY, United States; 10 Division of Nephrology, Department of Medicine, Taipei Veterans General Hospital, Taipei, Taiwan; 11 Center for Intelligent Drug Systems and Smart Bio-devices, National Yang Ming Chiao Tung University, Hsinchu, Taiwan; 12 Stem Cell Research Center, National Yang Ming Chiao Tung University, Taipei, Taiwan

**Keywords:** artificial intelligence, magnetic resonance imaging, MRI, shoulder, supraspinatus tendon tears

## Abstract

**Background:**

Supraspinatus tendon pathologies are common causes of shoulder pain. Magnetic resonance imaging (MRI) is the reference imaging method but requires expert interpretation. Automated classification may improve diagnostic consistency and support musculoskeletal imaging workflows.

**Objective:**

This study aimed to develop and evaluate a hierarchical deep learning model to classify supraspinatus tendon status as intact tendons, tendinopathy/partial-thickness tears, or full-thickness tears.

**Methods:**

A total of 1192 shoulder MRI scans were analyzed. The hierarchical system consisted of a left-right orientation classifier, a full-thickness tear detector (model F), and a classifier for distinguishing intact tendons from tendinopathy/partial-thickness tears (model ITP). A flat 3-class model served as a baseline comparator. Performance was evaluated on both an internal test set and an independent external cohort.

**Results:**

On the internal test set, the hierarchical system achieved a system-level sensitivity of 68.1% for tendinopathy/partial-thickness tears, outperforming the flat baseline (57.4%) while maintaining comparable sensitivity for full-thickness tears (hierarchical vs flat: 94.1% vs 95.1%). On the independent external cohort, the sensitivity for tendinopathy/partial-thickness tears was 45.5% for the hierarchical model and 18.2% for the flat baseline. The hierarchical model also showed a numerically higher balanced accuracy (hierarchical vs flat: 68.1% vs 64.5%), macro *F*_1_-score, and macro area under the curve, although its overall accuracy was lower (76.4% vs 79.8%).

**Conclusions:**

A hierarchical deep learning approach that mirrors clinical diagnostic reasoning may improve the recognition of tendinopathy and partial-thickness tears, a challenging category for nonspecialist readers. Given the overlapping CIs, these findings should be interpreted as indicative of a trend rather than definitive improvement. External validation supports feasibility across different MRI sources, though the predominance of data from a single institution limits generalizability and warrants further prospective evaluation.

## Introduction

Supraspinatus tendon abnormalities, including tendinopathy and partial- and full-thickness tears, refer to rotator cuff disorders resulting from stress injury, overuse, or age-associated changes. These rotator cuff disorders are leading causes of shoulder pain and functional limitation, affecting approximately 20% of the general population at some point during their lifetime [1]. Among the 4 rotator cuff tendons, the supraspinatus tendon is the most frequently affected, particularly in older adults [2]. Epidemiological studies suggest that nearly half of adults aged older than 60 years exhibit at least partial-thickness supraspinatus tears [3,4]. Supraspinatus tendon abnormalities have a profound impact on patients’ quality of life and the use of health care resources [5].

Magnetic resonance imaging (MRI), particularly with coronal T2-weighted sequences, is widely regarded as the imaging modality of choice for evaluating rotator cuff pathology [6]. Its superior soft-tissue contrast allows detailed visualization of tendon integrity, tear morphology, and associated joint abnormalities [7]. Clinicians rely heavily on MRI findings to assess the presence, extent, and severity of supraspinatus tendon pathology to directly inform treatment decisions. While full-thickness tears often necessitate surgical intervention to restore shoulder biomechanics, conservative measures (eg, physical therapy, modifying physical activity, and corticosteroid injections) are typically used to manage tendinopathy and partial-thickness tears [8].

Artificial intelligence (AI), particularly deep learning techniques, can automate complex pattern recognition tasks and support radiologic decision-making [9,10]. However, many early AI models for rotator cuff evaluation lacked demonstrated generalizability, primarily because they were trained on single-institution datasets or homogeneous imaging protocols [11,12]. In addition, prior studies varied considerably in class definitions, input strategies (eg, single-plane vs multisequence MRI), and imaging vendor characteristics, further limiting the comparability of the results and the translation of these models into real-world clinical practice [11,13]. Additionally, clinician trust and adoption of these early AI models remain limited, as their interpretability has often been insufficient to assure physicians that model decisions align with established radiological and anatomical principles.

To address the clinical challenge of accurately identifying supraspinatus tendon pathologies on shoulder MRI, we constructed a hierarchical 3D deep learning model based on the ResNet-18 architecture. The model was trained on a dataset comprising coronal T2-weighted MRI scans, and it classifies tendon status into the following three clinically relevant categories: (1) intact tendons, (2) tendinopathy or partial-thickness tears, and (3) full-thickness tears. To enhance interpretability and support clinicians’ trust, Score-weighted Class Activation Mapping (Score-CAM) was integrated to visualize the imaging features that drive model predictions. The robust and interpretable AI framework was designed to support treatment decisions and facilitate real-world integration of deep learning tools in musculoskeletal imaging.

## Methods

### Ethical Considerations

The protocol of this retrospective study was approved by the Institutional Review Board of Taipei Veterans General Hospital (TVGH; IRB number: 2023-01-024AC). The requirement for informed consent was waived owing to the retrospective nature of the study and the use of deidentified imaging data. All procedures were conducted in accordance with the ethical standards of the institutional research committee and the principles outlined in the Declaration of Helsinki. The study also complied with relevant national legislation and institutional guidelines governing human subject research. Participants did not receive any compensation for this study.

### Patient Selection Criteria and Dataset

Coronal T2-weighted shoulder MRI images from 1192 cases were collected for training the new AI-based hierarchical model developed in this study. These images were predominantly obtained from TVGH, but the dataset also included images collected across 65 institutions or uploaded from external facilities for second-opinion evaluations.

Patients with tumors, prior shoulder surgery, humeral head osteonecrosis, moderate to severe osteoarthritis (Kellgren-Lawrence [KL] grades 2 to 4), or a history of fractures around the shoulder were excluded. KL grading was assessed using anteroposterior shoulder radiographs, based on osteophyte formation and joint space narrowing in accordance with established definitions. The distinction between KL grades 1 and 2 followed the original criteria, with definite osteophyte formation serving as the primary threshold for grade 2 [14]. Based on this definition, patients with KL grade ≥2 were excluded to minimize potential confounding from glenohumeral degenerative changes. In this study, KL grading was applied as a pragmatic radiographic exclusion criterion rather than for formal characterization of shoulder osteoarthritis. Although originally developed for other joints, KL grading has been applied in prior radiographic and population-based studies of glenohumeral osteoarthritis, with demonstrated interobserver reliability [15,16].

Cases were categorized into the following 3 groups on the basis of supraspinatus tendon status: intact tendons (n=449), tendinopathy or partial-thickness tears (n=235), and full-thickness tears (n=508). The reference standard for labeling intact tendons and tendinopathy/partial-thickness tears was based on MRI interpretations by consensus between 1 musculoskeletal radiologist and 2 orthopedic surgeons, each with over 10 years of specialty training experience. Full-thickness tears were confirmed through consistent MRI findings and verification by arthroscopic surgical reports.

Of note, tendinopathy and partial-thickness tears were grouped into a single category based on both clinical and methodological considerations. Structurally, tendon continuity is preserved in both these conditions, in contrast to full-thickness tears, which disrupt tendon integrity. Clinically, the management of tendinopathy and partial-thickness tears is similar, with conservative treatment (rest, rehabilitation, medication, or injections) typically preferred before considering surgical intervention. Moreover, the imaging findings of tendinopathy and early partial-thickness tears often overlap, and interobserver agreement in distinguishing them can be limited [17]. Combining these 2 entities therefore reduces diagnostic variability and aligns more closely with clinical practice.

### Definitions

**Figure 1.**
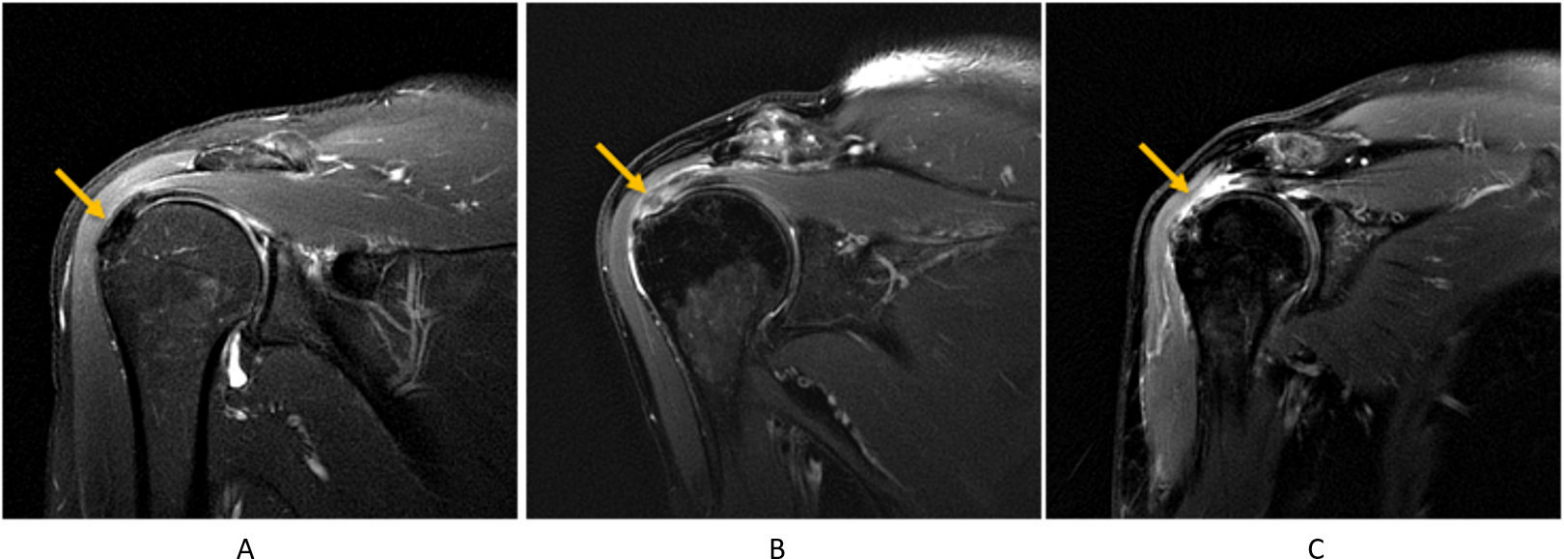
Illustration of supraspinatus tendon conditions: intact tendons (A), tendinopathy/partial-thickness tears (B), and full-thickness tears (C).

Figure 1 presents the different supraspinatus tendon pathologies, including intact tendons, tendinopathy/partial-thickness tears, and full-thickness tears.

MRI-based diagnostic criteria are as follows: tendinopathy is defined by tendon thickening with an inhomogeneous signal intensity and increased signal across all pulse sequences; partial-thickness tears are defined by fluid signal intensity partially filling a gap within the tendon, most evident on fat-suppressed T2-weighted images; and full-thickness tears are defined by complete disruption of the tendon, appearing as high signal intensity spanning the entire tendon thickness on all pulse sequences [18,19].

### Dataset Splitting

Cases from each year between 2018 and 2024 were stratified by acquisition year and randomly assigned at the patient level to a training set (64%), a validation set (16%), and an internal test set (20%), ensuring that the year-wise distribution was preserved across all subsets.

The training set consisted of 287 intact tendon cases, 150 tendinopathy or partial-thickness tear cases, and 327 full-thickness tear cases. The validation set included 72 intact tendon cases, 38 tendinopathy or partial-thickness tear cases, and 79 full-thickness tear cases. The internal test set comprised 90 intact tendon cases, 47 tendinopathy or partial-thickness tear cases, and 102 full-thickness tear cases.

The training, validation, and internal test sets demonstrated similar mean echo time and repetition time values. Age and sex distributions were similar across the training, validation, and internal test sets. The distributions of shoulder side and tear type were also comparable among the datasets (Table 1).

**Table 1.** Data characteristics (case level).

Variable^a^	Training set (n=764)	Validation set (n=189)	Internal set (n=239)	External set (n=89)
Acquisition parameters				
TE^b^ (ms), mean (SD)	62.31 (13.23)	61.87 (14.26)	60.54 (15.72)	74.59 (20.79)
TR^c^ (ms), mean (SD)	3310.85 (674.16)	3258.58 (714.70)	3207.26 (771.03)	4546.19 (1961.84)
Acquisition timeframe	01-26-2018 to 05-19-2024	02-14-2018 to 03-28-2024	02-14-2018 to 05-19-2024	02-13-2020 to 04-10-2024
Demographic characteristics and dataset composition				
Age (years), mean (SD)	55.86 (14.61)	55.03 (14.39)	56.91 (15.08)	53.84 (15.37)
Sex, n (%)				
Female	367 (48.0)	99 (52.4)	114 (47.7)	38 (42.7)
Male	397 (52.0)	90 (47.6)	125 (52.3)	51 (57.3)
Shoulder side, n (%)				
Right	504 (66.0)	124 (65.6)	158 (66.1)	57 (64.0)
Left	260 (34.0)	65 (34.4)	81 (33.9)	32 (36.0)
Tear type, n (%)				
Intact tendons	287 (37.6)	72 (38.1)	90 (37.7)	35 (39.3)
Tendinopathy/partial-thickness tears	150 (19.6)	38 (20.1)	47 (19.7)	11 (12.4)
Full-thickness tears	327 (42.8)	79 (41.8)	102 (42.7)	43 (48.3)
Manufacturer, n (%)				
Canon Medical Systems	1 (0.1)	0 (0.0)	0 (0.0)	2 (2.2)
GE Medical Systems	593 (77.6)	137 (72.5)	174 (72.8)	45 (50.6)
Hitachi Medical Corporation	7 (0.9)	2 (1.1)	2 (0.8)	1 (1.1)
Philips Medical Systems	83 (10.9)	30 (15.9)	36 (15.1)	38 (42.7)
Siemens	77 (10.1)	18 (9.5)	27 (11.3)	2 (2.2)
Toshiba	3 (0.4)	2 (1.1)	0 (0.0)	1 (1.1)

a Magnetic resonance imaging acquisition parameters and demographic information were extracted from DICOM metadata, and left-right shoulder labels were manually assigned at the case level.

b TE: echo time.

c TR: repetition time.

### Imaging Characteristics

A total of 1192 cases were collected, and the internal cohort was predominantly obtained from TVGH (987 cases; Table 2).

For the internal cohort, the dataset included MRI scans acquired using 29 scanner models from the following 6 vendors: Canon Medical Systems (Ōtawara-shi, Tochigi, Japan), GE Healthcare (Chicago, IL), Philips Medical Systems (Andover, MA; headquartered in Amsterdam, Netherlands), Siemens Healthineers (Forchheim, Germany), Hitachi Medical Corporation (Tokyo, Japan), and Toshiba Medical Systems (Ōtawara-shi, Tochigi, Japan) (Table 1).

The in-plane resolution (X-Y pixel spacing) ranged from approximately 0.19 mm to 0.86 mm, with the majority of cases falling within the range of 0.30‐0.39 mm. Within this range, 0.3125 mm was the most frequently observed in-plane resolution (496 cases). Slice spacing ranged from 3.0 mm to 5.0 mm, with 3.5 mm being the most commonly used spacing. Slice thickness varied from 2.0 mm to 4.5 mm, and the most common thickness was 3.0 mm. The detailed distributions of slice thickness, slice spacing, and in-plane resolution across the training, validation, and internal test sets are summarized in Tables S1 and S2 in Multimedia Appendix 1.

**Table 2.** Top 5 sites (by total cases) contributing to the internal cohort.

Institution	Training set, n	Validation set, n	Internal set, n	Total, n
Taipei Veterans General Hospital	640	155	192	987
Cheng Hsin General Hospital	36	12	17	65
Kinmen Hospital of the Ministry of Health and Welfare	12	4	5	21
Mackay Memorial Hospital Taipei	3	2	1	6
Postal Hospital	3	1	1	5

### External Validation Set

An independent external validation dataset was created from institutions not included in the model development cohort to evaluate the generalizability of the proposed model.

A total of 89 cases were collected from 12 medical institutions across Taiwan, with the largest contribution from Taoyuan Hospital, Ministry of Health and Welfare (37 cases; Table 3). The external validation set comprised 35 intact tendon cases, 11 tendinopathy or partial-thickness tear cases, and 43 full-thickness tear cases.

None of the external cases were used during model training, validation, or hyperparameter tuning (Table 1).

**Table 3.** Top 5 sites (by total cases) contributing to the external cohort.

Institution	External set, n
Taoyuan Hospital, Ministry of Health and Welfare	37
Taitung Hospital, Ministry of Health and Welfare	20
China Medical University Hsinchu Hospital	10
Sinwu Branch, Taoyuan Hospital, Ministry of Health and Welfare	8
Taoyuan Armed Forces General Hospital Hsinchu Branch	5

### Data Preprocessing

#### Overview

All DICOM images were first converted to NIfTI format, followed by preprocessing and model input preparation. All MRI volumes were visually inspected to confirm the absence of burned-in text or laterality markers. No field-of-view cropping or masking was applied to remove such information, ensuring that the model did not rely on textual artifacts for laterality classification.

To ensure anatomical consistency, all MRI images of left shoulders were horizontally flipped so that all cases involved right shoulders, thereby reducing directional variability and improving model robustness. Pixel intensities were normalized to the range [0, 1] using linear minimum-maximum scaling on a per-volume basis.

To standardize volumetric input across heterogeneous MRI acquisitions, all scans were resampled using linear interpolation to a uniform spatial resolution, with an in-plane voxel spacing of 0.3125×0.3125 mm and a slice thickness of 3 mm.

Each case was then standardized to 16 slices by extracting the central coronal region of the resampled volume. For cases with fewer than 16 slices, linear interpolation along the slice direction was applied to generate intermediate slices, avoiding zero-padding and preserving anatomical continuity. Because field-of-view centering may vary across scanners and technicians, all cases were reviewed on a case-by-case basis to confirm that the supraspinatus tendon and relevant pathology were present within the selected slice range.

Each input sample was formatted as a 3×L×H×W tensor, where L denotes the number of slices, H represents image height, and W represents image width. All slices were resized to 256×256 pixels using bilinear interpolation. As the original MRI data were grayscale, intensity values were replicated across 3 channels to ensure compatibility with pretrained convolutional neural network (CNN) architectures.

#### Normalization

We applied several normalization strategies aimed at reducing input variability and simplifying the learning task to improve model performance and generalizability. These preprocessing techniques were based on the dynamic range and statistical distribution of the MRI input data to ensure consistent model performance across diverse acquisition settings.

#### Interpolation

Because the number of slices varied across MRI scans, all inputs were standardized to 16 slices by selecting the central portion of each scan. For cases with fewer than 16 slices, linear interpolation was applied along the z-axis to generate intermediate slices, avoiding the use of zero-padding, which can introduce artificial discontinuities.

The interpolation formula was defined as follows. To estimate the voxel intensity at a target position z, located between 2 known slices z0 and z1, with voxel intensities V(x,y,z0) and V(x,y,z1), we used the following formula:


$$V(x,y,z)=(1-w_{z})\;V(x,y,z_{0})+w_{z}\;V(x,y,z_{1})$$

where wz = (z−z0)/(z1−z0).

#### Spacing and Slice Thickness

The in-plane resolution (X and Y spacing) across the dataset ranged from 0.192 mm to 0.781 mm. The most common resolution was 0.3125×0.3125 mm, observed in 765 cases. Slice spacing (interslice distance) ranged from 3 mm to 5 mm, with 3.5 mm being the most frequently used value (574 cases). Slice thickness varied from 2 mm to 4.5 mm, with 3 mm being the most common setting, found in 958 cases.

All MRI scans were normalized to a standard voxel spacing of 0.3125×0.3125 mm in the X and Y dimensions and 3 mm in the Z dimension in order to reduce the variability introduced by heterogeneous acquisition parameters. Standardizing Z-spacing to match slice thickness also preserved greater anatomical detail, which is critical for identifying subtle tear features. Linear resampling implemented via the SimpleITK library was used for interpolation, allowing precise adjustment of voxel spacing in the NIfTI-formatted MRI data (Table S1 in Multimedia Appendix 1)

#### Left-Right Shoulder Classification

In clinical practice, surgeons are able to diagnose shoulder injuries regardless of whether the image is mirrored; however, deep learning models may treat left and right shoulder pathologies as distinct owing to asymmetrical spatial features, which may hinder model generalization. To address this, we trained a left-right model to identify shoulder laterality, and cases predicted as left (406/1192) were horizontally flipped so that all inputs were standardized as right shoulders.

Although DICOM metadata may contain laterality information, it was not consistently available or reliable across datasets from different institutions. In some cases, laterality tags were missing, incomplete, or inconsistent with the actual image orientation. Therefore, we adopted an image-based classification approach to ensure robust and standardized determination of shoulder laterality across all inputs.

This classifier used a 3D CNN architecture implemented using torchvision, consistent with the backbone used in subsequent models. The input for the classifier was the entire MRI volume at the case level, and the output was a binary prediction indicating left or right shoulder orientation.

Ground-truth labels were manually annotated based on image orientation and acquisition metadata. All left shoulder volumes were subsequently mirrored to match the right shoulder orientation, thereby reducing directional variability in the training data.

### Model Architecture and Training

**Figure 2.**
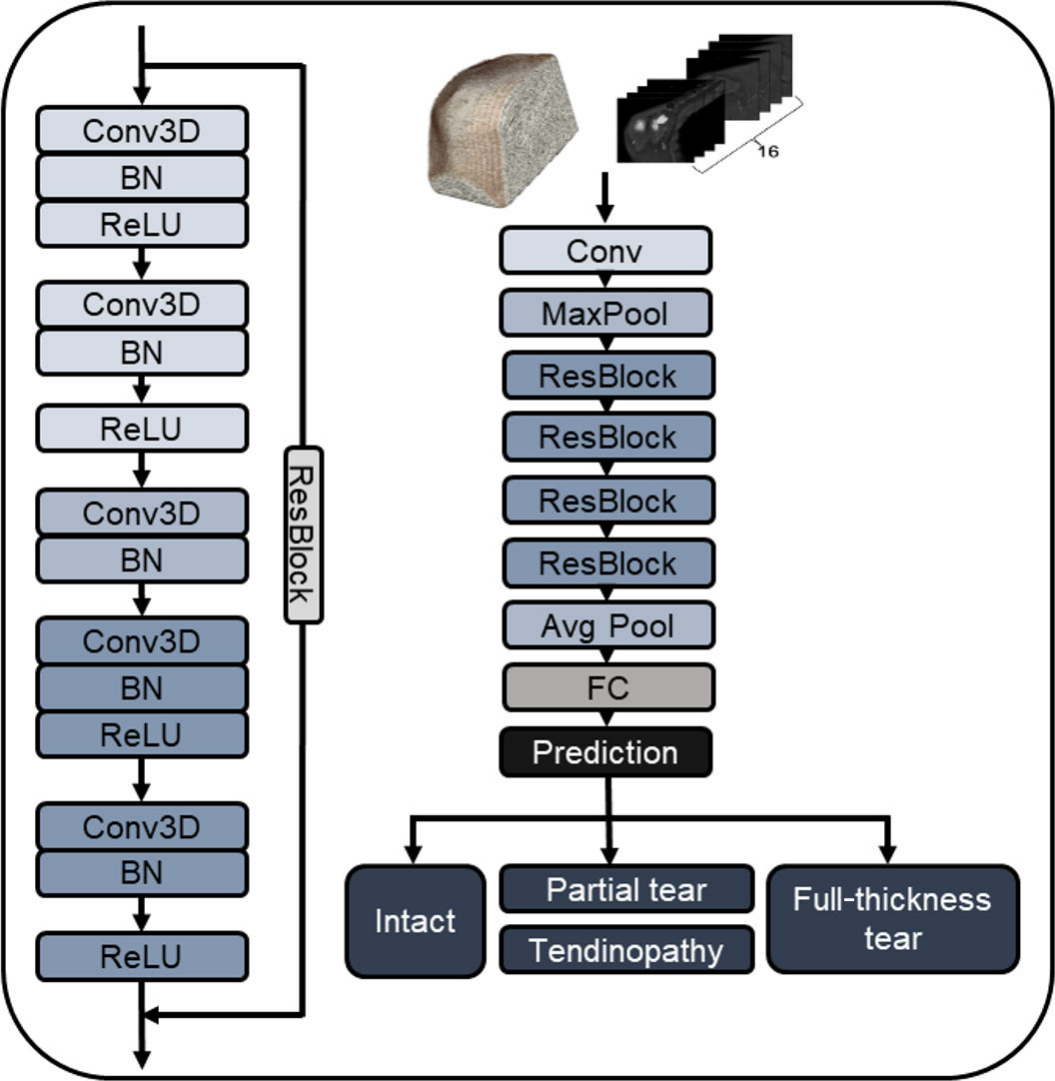
Architecture of the 3D ResNet-18 model used in this study. BN: batch normalization; Conv: convolutional layer; Conv3D: 3D convolutional layer; FC: fully connected layer; MaxPool: max pooling; ReLU: rectified linear unit; ResBlock: residual block.

A 3D ResNet-18 CNN was constructed for classification. The model architecture is a spatiotemporal extension of the conventional ResNet framework, incorporating residual connections and 3D convolutions to process volumetric data effectively [20]. The 3D ResNet-18 model used in this study was based on the torchvision library implementation within the PyTorch framework (Figure 2). The model used standard BatchNorm3d layers as implemented in the default architecture.

Using transfer learning, the model was initialized with pretrained weights, and the final fully connected layer was modified to perform 2-class classification for each task. During training, a validation set was used to monitor performance and select the best-performing model. Score-CAM was used to generate heatmaps for model interpretability, highlighting the most influential regions in model predictions.

### Hierarchical Classification Framework

**Figure 3.**
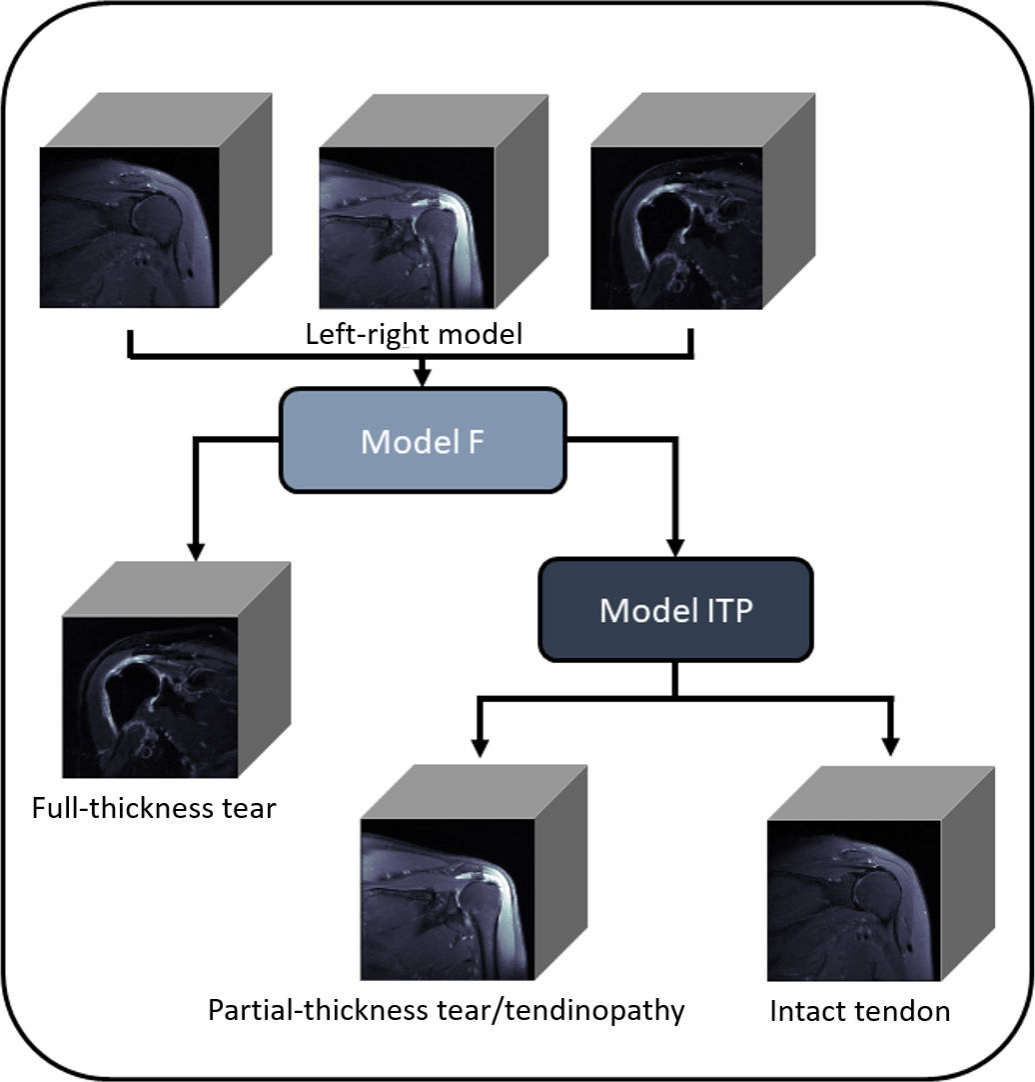
Hierarchical model architecture for 3-class classification of supraspinatus tendon status. Model F: classifier for differentiating full-thickness tears from non–full-thickness cases (intact tendons or tendinopathy/partial-thickness tears); model ITP: classifier for distinguishing intact tendons from tendinopathy/partial-thickness tears.

To achieve 3-class classification, a hierarchical model composed of 3 sequential 3D ResNet-18 classifiers was designed as follows (Figure 3):

- Model F: Differentiated full-thickness tears from non–full-thickness cases (intact tendons or tendinopathy/partial-thickness tears).
- Model ITP: Applied only to non–full-thickness cases; this model distinguished intact tendons from tendinopathy/partial-thickness tears.

The use of this stepwise approach helped reduce classification complexity and improve overall performance by leveraging domain knowledge of clinical relevance.

Although the final prediction was reported as a 3-class outcome (intact tendons, tendinopathy/partial-thickness tears, and full-thickness tears), the proposed framework performed hierarchical inference through 2 sequential binary classifiers. Specifically, the first-stage model distinguished full-thickness tears from non–full-thickness cases, and only cases predicted as non–full-thickness were subsequently passed to the second-stage model to differentiate intact tendons from tendinopathy/partial-thickness tears. Therefore, the reported 3-class confusion matrices reflected the final outputs of a hierarchical decision process rather than a flat 3-class classifier.

In addition, for probabilistic evaluation (receiver operating characteristic [ROC] curves and macro area under the curve [AUC]), we derived a unified 3-class probability distribution using conditional probability decomposition. Specifically, the predicted conditional probabilities were defined as follows:

- Model F outputs: *P* (non–full-thickness∣𝑥), *P* (full-thickness∣𝑥)
- Model ITP outputs: *P* (intact∣non–full-thickness, 𝑥), *P* (ITP∣non–full-thickness, 𝑥)

where ITP denotes tendinopathy/partial-thickness tears.

Final 3-class probabilities were computed using conditional probability decomposition as follows:

*P* (intact∣𝑥) = *P* (non–full-thickness∣𝑥)⋅ *P* (intact∣non–full-thickness, 𝑥)

*P* (ITP∣𝑥) = *P* (non–full-thickness∣𝑥)⋅ *P* (ITP∣non–full-thickness, 𝑥)

*P* (full-thickness∣𝑥) = *P* (full-thickness∣𝑥)

where probabilities from model F and model ITP were obtained via Softmax normalization of the corresponding logits. This formulation ensures that the final outputs form a valid probability distribution that sums to 1.

These derived probabilities were used to compute one-vs-rest ROC curves and macro AUC for the 3-class classification task.

### Class Imbalance Handling

The dataset exhibited class imbalance across diagnostic categories, particularly for partial-thickness tears and tendinopathy. In the proposed hierarchical framework, class weighting, a strategy shown by Buda et al [21] to be effective and stable for handling imbalanced medical imaging datasets in CNNs, was applied only to the second-stage classifier (model ITP), which distinguishes intact tendons from partial-thickness tears and tendinopathy.

This design choice was motivated by the observation that class imbalance and visual ambiguity are most pronounced at this stage. In contrast, the first-stage classifier (model F), which detects full-thickness tears, targets a clinically distinct and more visually separable category. Selective application of class weighting therefore allowed us to improve sensitivity for the most challenging minority class without destabilizing optimization in the first-stage model as follows:


$$\text{Class Weight}(i)=\frac{N_{\text{samples}}}{N_{\text{classes}}\cdot N_{i}}$$

### Model Interpretability: Score-CAM

To visualize the regions of interest that contributed most to model decisions, Score-CAM was implemented [22]. Unlike gradient-based methods, such as Gradient-weighted Class Activation Mapping [23], Score-CAM assesses the contribution of each activation map by forwarding masked images and observing their effect on the model’s output rather than relying on backpropagated gradients. This method provides intuitive, spatially localized visualizations of model attention to support interpretability. The evaluation of these visualizations was qualitative and based on representative cases, without formal quantitative assessment or radiologist-based validation (Figures S1-3 in Multimedia Appendix 2).

### Deep Learning Experiment

#### System and Software

All models were trained on a workstation equipped with an NVIDIA GeForce RTX 3060 GPU (12 GB VRAM), a 12th Generation Intel Core i7-12700F CPU, and 64 GB of RAM, running Ubuntu 22.04.1 LTS. Python 3.10.12 and PyTorch 2.4.0 were used to create a stable and compatible environment for developing and running PyTorch-based applications.

#### Preprocessing Ablation Experiments

To quantify the impact of individual preprocessing components, we conducted a series of controlled ablation experiments using model ITP as a fixed evaluation framework. The network architecture, dataset split, training procedure, and evaluation metrics were held constant across all experiments, while preprocessing strategies were selectively varied.

The following three preprocessing factors were evaluated: (1) left-right shoulder mirroring, (2) slice standardization strategy (zero-padding vs linear interslice interpolation to 16 slices), and (3) voxel spacing normalization.

Model ITP was selected for these ablation experiments because it provided a stable and representative architecture for assessing preprocessing effects without confounding architectural changes. The final preprocessing pipeline, identified through this ablation analysis, was subsequently applied consistently to both model ITP and model F to ensure methodological uniformity across experiments.

#### Model Architecture, Pretrained Weights, and Transfer Learning

All experiments were conducted using 3D CNNs based on the 3D ResNet-18 architecture implemented in the torchvision library within the PyTorch framework. Model weights were initialized using parameters pretrained on the Kinetics-400 dataset. The pretrained weights were applied to all convolutional layers, while the final fully connected layers were randomly initialized and trained from scratch for the target classification tasks.

#### Training Strategy and Class Imbalance Handling

A transfer learning strategy was adopted for all models. For the hierarchical framework, the final fully connected layers were adapted for binary classification in each subtask.

To address class imbalance, class-weighted cross-entropy loss was applied only to the second-stage classifier (model ITP), which distinguishes intact tendons from partial-thickness tears or tendinopathy. Class weights were computed from the training set using the balanced weighting scheme provided by the scikit-learn library, ensuring that underrepresented classes contributed proportionally to the optimization objective.

In contrast, no class weighting was applied to the first-stage classifier (model F), which focuses on detecting full-thickness tears—a clinically distinct category with more separable imaging characteristics.

#### Hyperparameters and Optimization

Optimal hyperparameters were identified through empirical tuning. A learning rate of 1×10⁻⁵ and a batch size of 8 were selected, as these settings provide stable convergence while maintaining feasible training efficiency for 3D volumetric models. Model parameters were optimized using the Adam optimizer.

#### Model Selection and Evaluation

The best-performing model was selected based on validation macro-averaged sensitivity (macro sensitivity), which is less affected by class imbalance than overall accuracy. Final performance was evaluated on independent internal and external test sets using accuracy, balanced accuracy, macro-averaged AUC, and confusion matrices.

### Flat 3-Class Baseline

For comparison, we trained a conventional flat 3-class classifier to predict intact tendons, tendinopathy/partial-thickness tears, and full-thickness tears directly. This model used the same preprocessing pipeline as the hierarchical framework, including left-right shoulder classification for anatomical standardization, followed by selection of the central 16 slices, resizing, and intensity normalization. The same 3D ResNet-18 backbone, training-validation-test splits, and optimization settings were applied.

To mitigate class imbalance, class-weighted cross-entropy loss was used during training, with class weights computed from the training set using the balanced weighting scheme provided by the scikit-learn library.

The only architectural difference was that the flat baseline used a single 3-class Softmax output layer, replacing the 2-stage hierarchical design.

### Computational Cost

All models were trained for 1000 epochs on a single GPU. Because wall-clock training time was not explicitly recorded during the full training process, GPU training time was measured over 10 epochs using identical hardware, batch size, and training settings. The total training time for 1000 epochs was then extrapolated assuming linear scaling with respect to the number of epochs.

### Evaluation Metrics

For multiclass classification, precision, *F*_1_-score, sensitivity, and specificity were computed using macro-averaging, whereby each class (intact tendons, tendinopathy/partial-thickness tears, and full-thickness tears) contributes equally regardless of class prevalence.

ROC AUC values were computed using a one-vs-rest strategy from Softmax probability outputs and have been reported as macro-averaged AUC values. Overall accuracy corresponded to micro-averaged performance.

## Results

### Preprocessing Ablation Experiments

The impact of different preprocessing strategies on model performance was evaluated using model ITP under a controlled ablation framework. Balanced accuracy (macro-averaged sensitivity) was used as the primary evaluation metric to account for class imbalance. The network architecture, dataset split, and training protocol were identical across all experiments, with only preprocessing components being varied.

As summarized in Table 4, applying interpolation-based slice standardization and voxel spacing normalization without left-right mirroring resulted in a balanced accuracy of 0.815. Introducing left-right mirroring while using zero-padding to standardize slice count yielded a modest increase in balanced accuracy to 0.820. This improvement suggests that anatomical orientation alignment reduces directional variability; however, the use of padding may introduce nonphysiological discontinuities along the slice dimension.

**Table 4.** Balanced accuracy of model ITP^a^ under different preprocessing strategies.

Preprocessing configuration	Balanced accuracy
No mirroring, interpolation to 16 slices, with spacing normalization	0.815
With mirroring, padding to 16 slices, with spacing normalization	0.820
With mirroring, interpolation to 16 slices, without spacing normalization	0.799
With mirroring, interpolation to 16 slices, with spacing normalization	0.826

a Model ITP: classifier for distinguishing intact tendons from tendinopathy/partial-thickness tears.

When left-right mirroring and interpolation were applied without voxel spacing normalization, balanced accuracy decreased to 0.799. This reduction indicates that heterogeneity in acquisition parameters, including in-plane resolution and slice spacing, negatively affects model robustness when spatial normalization is omitted.

The highest balanced accuracy (0.826) was achieved when left-right mirroring, interpolation-based slice standardization, and voxel spacing normalization were jointly applied. Based on these findings, the full preprocessing pipeline was selected for subsequent experiments and applied consistently to both model ITP and model F.

### Model Training and Performance

All models were trained for 1000 epochs on a single GPU. As wall-clock time was not recorded for the full training run, runtime was measured over 10 epochs using end-to-end wall-clock timing (including both training and validation phases, as well as data loading and evaluation overhead) and then extrapolated to 1000 epochs assuming linear scaling.

Based on this estimation, the total end-to-end GPU runtime for 1000 epochs was approximately 41.9 hours for the left-right classifier, 72.5 hours for model F, and 22.4 hours for model ITP (Table 5). These values should be interpreted as estimated runtimes, as linear extrapolation may not fully capture potential variability in longer training runs (Table 5).

**Table 5.** Estimated end-to-end GPU runtime (including training and validation) based on linear extrapolation from 10 epochs.

Model	10 epochs	1000 epochs
Left-right classifier	25.15 min	2515 min (approximately 41.9 h)
Model F^a^	43.51 min	4351 min (approximately 72.5 h)
Model ITP^b^	13.46 min	1346 min (approximately 22.4 h)

a Model F: classifier for differentiating full-thickness tears from non–full-thickness cases (intact tendons or tendinopathy/partial-thickness tears).

b Model ITP: classifier for distinguishing intact tendons from tendinopathy/partial-thickness tears.

Shoulder laterality (left or right) was determined after first training a binary classifier. The model achieved perfect validation performance (100%) by epoch 2.

Model F was then trained to differentiate full-thickness tears from non–full-thickness cases. The highest validation-balanced accuracy of 96.74% was achieved at epoch 93. Finally, model ITP was trained to distinguish intact tendons from cases with tendinopathy or partial-thickness tears. This model reached its best validation-balanced accuracy of 87.10% at epoch 983.

Training progress and performance metrics across epochs for each model are presented in Figure S4 in Multimedia Appendix 2.

### Internal Test Set Performance

Model F, designed to differentiate full-thickness tears from non–full-thickness cases, achieved an accuracy of 93.7% on the internal validation set. The model demonstrated a macro precision of 93.5% and a macro *F*_1_-score of 93.6%. The sensitivity for full-thickness tears was 94.1%, and the specificity for non–full-thickness cases was 93.4%. The ROC AUC was 0.984.

Model ITP, which further classified non–full-thickness cases into intact tendons or tendinopathy/partial-thickness tears, achieved an accuracy of 82.6%. The model yielded a macro precision of 80.8% and a macro *F*_1_-score of 81.3%. The sensitivity for tendinopathy/partial-thickness tears was 83.0%, while the specificity for intact tendons was 82.3%. The ROC AUC was 0.904.

**Figure 4.**
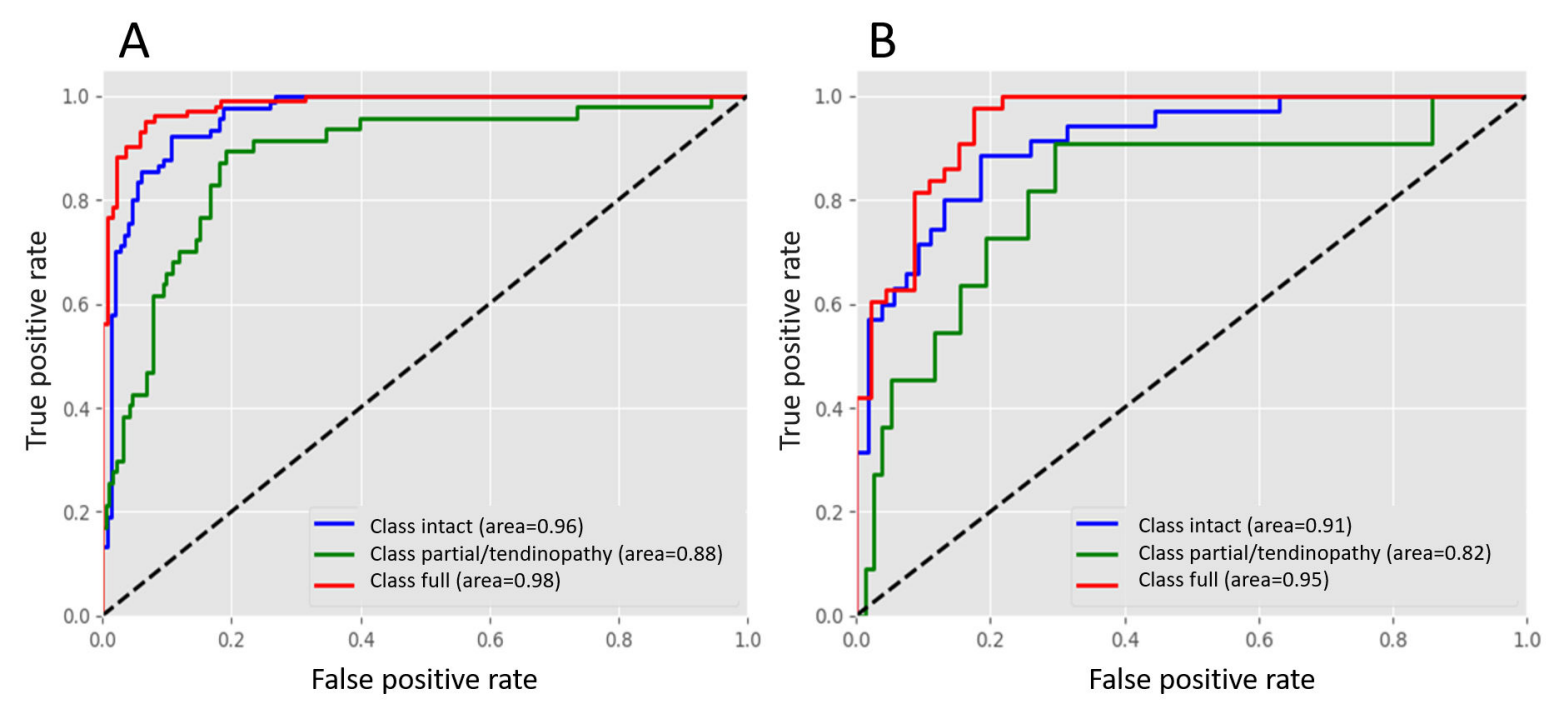
Multiclass receiver operating characteristic (ROC) curves: (A) internal validation set and (B) external validation set.

The hierarchical model, which used the left-right model for preprocessing and integrated outputs from model F and model ITP for the final 3-class classification, achieved an accuracy of 84.1% and a macro-average AUC of 0.942. The macro sensitivity and macro specificity were 81.0% and 92.4%, respectively. In addition, the model yielded a macro precision of 80.7% and a macro *F*_1_-score of 80.6% (Figure 4A; Tables6^8^).

**Table 6.** Performance of hierarchical and flat classification models on the internal and external test sets.

Dataset and model	Accuracy, mean (95% CI^a^)	Macro^b^ AUC^c^^,^^d^, mean (95% CI^a^)	Macro^b^ sensitivity, mean (95% CI^a^)	Macro^b^ specificity, mean (95% CI^a^)	Macro^b^ precision, mean (95% CI^a^)	Macro^b^ *F*_1_-score, mean (95% CI^a^)
Internal test set (n=239)						
Flat	0.816 (0.770‐0.862)	0.935 (0.907‐0.959)	0.771 (0.715‐0.827)	0.907 (0.884‐0.929)	0.781 (0.724‐0.836)	0.773 (0.716‐0.828)
Hierarchical	0.841 (0.795‐0.883)	0.942 (0.914‐0.965)	0.810 (0.756‐0.864)	0.924 (0.902‐0.944)	0.807 (0.756‐0.857)	0.806 (0.754‐0.857)
External test set (n=89)						
Flat	0.798 (0.730‐0.854)	0.868 (0.789‐0.939)	0.645 (0.561‐0.740)	0.884 (0.846‐0.918)	0.688 (0.525‐0.882)	0.645 (0.541‐0.762)
Hierarchical	0.764 (0.685‐0.843)	0.893 (0.823‐0.949)	0.681 (0.565‐0.797)	0.877 (0.832‐0.919)	0.683 (0.572‐0.786)	0.674 (0.560‐0.782)

a 95% CIs were estimated using stratified bootstrap resampling (2000 iterations).

b Macro-averaged metrics assign equal weight to each class (intact tendons, tendinopathy/partial-thickness tears, and full-thickness tears).

c AUC: area under the curve.

d AUC values were computed using Softmax probability outputs with a one-vs-rest strategy.

**Table 7.** Hierarchical and flat 3-class classification confusion matrix.

Dataset, model, and class	Intact tendons, n^a^	Tendinopathy/partial-thickness tears, n^a^	Full-thickness tears, n^a^
Internal test set (n=239)			
Hierarchical classification^b^			
Intact tendons	73	16	1
Tendinopathy/partial-thickness tears	7	32	8
Full-thickness tears	1	5	96
Flat 3-class classification			
Intact tendons	71	15	4
Tendinopathy/partial-thickness tears	7	27	13
Full-thickness tears	2	3	97
External test set (n=89)			
Hierarchical classification^b^			
Intact tendons	23	6	6
Tendinopathy/partial-thickness tears	4	5	2
Full-thickness tears	1	2	40
Flat 3-class classification			
Intact tendons	27	2	6
Tendinopathy/partial-thickness tears	5	2	4
Full-thickness tears	0	1	42

a Raw case counts.

b Hierarchical classification confusion matrices summarize the final 3-class predictions produced by the hierarchical inference pipeline.

**Table 8.** The performance of the left-right model, model F^a^, and model ITP^b^ on the internal and external test sets.

Model and dataset	Accuracy, value (95% CI)	ROC AUC^c^, value (95% CI)	Precision, value (95% CI)	*F*_1_-score, value (95% CI)	Sensitivity, value (95% CI)	Specificity, value (95% CI)
Left-right model						
Internal	0.996 (0.987‐1.000)	1.000 (1.000‐1.000)	0.994 (0.982‐1.000)	0.995 (0.986‐1.000)	1.000 (1.000‐1.000)^d^	0.994 (0.981‐1.000)^e^
External	1.000 (1.000‐1.000)	1.000 (1.000‐1.000)	1.000 (1.000‐1.000)	1.000 (1.000‐1.000)	1.000 (1.000‐1.000)^d^	1.000 (1.000‐1.000)^e^
Model F (full-thickness tears vs non–full-thickness cases)						
Internal	0.937 (0.908‐0.967)	0.984 (0.971‐0.994)	0.935 (0.905‐0.964)	0.936 (0.905‐0.966)	0.941 (0.892‐0.980)^f^	0.934 (0.891‐0.971)^g^
External	0.877 (0.809‐0.944)	0.950 (0.902‐0.984)	0.883 (0.812‐0.944)	0.877 (0.807‐0.944)	0.930 (0.837‐1.000)^f^	0.828 (0.717‐0.935)^g^
Model ITP (intact tendons vs tendinopathy/partial-thickness tears)						
Internal	0.826 (0.759‐0.883)	0.904 (0.843‐0.954)	0.808 (0.741‐0.870)	0.813 (0.743‐0.875)	0.830 (0.723‐0.936)^h^	0.823 (0.744‐0.900)^i^
External	0.762 (0.630‐0.870)	0.815 (0.629‐0.958)	0.694 (0.556‐0.835)	0.698 (0.551‐0.839)	0.640 (0.364‐0.909)^h^	0.800 (0.657‐0.914)^i^

a Model F: classifier for differentiating full-thickness tears from non–full-thickness cases (intact tendons or tendinopathy/partial-thickness tears).

b Model ITP: classifier for distinguishing intact tendons from tendinopathy/partial-thickness tears.

c ROC AUC: receiver operating characteristic area under the curve.

d Data for left.

e Data for right.

f Data for full-thickness tears.

g Data for non–full-thickness cases.

h Data for tendinopathy/partial-thickness tears.

i Data for intact tendons.

### External Dataset Performance

On the external validation set, the left-right model maintained perfect performance, achieving 100% accuracy, precision, sensitivity, and specificity, and an ROC AUC of 1.000, confirming strong generalizability for shoulder laterality identification.

Model F achieved an accuracy of 87.7% on the external validation set. The model demonstrated a macro precision of 88.3% and a macro *F*_1_-score of 87.7%. The sensitivity for full-thickness tears was 93.0%, while the specificity for non–full-thickness cases was 82.8%. The ROC AUC was 0.95, indicating good discrimination despite reduced performance compared with internal validation.

Model ITP showed increased performance variability on the external dataset, achieving an accuracy of 76.2%. The macro precision and macro *F*_1_-score were 69.4% and 69.8%, respectively. The sensitivity for tendinopathy/partial-thickness tears was 64.0%, while the specificity for intact tendons was 80.0%. The ROC AUC was 0.815.

The hierarchical model, which used the left-right model for preprocessing and integrated outputs from model F and model ITP for the final 3-class classification, achieved an accuracy of 76.4% and a macro-average AUC of 0.893. The corresponding macro sensitivity, macro specificity, macro precision, and macro *F*_1_-score were 68.1%, 87.7%, 68.3%, and 67.4%, respectively (Figure 4B; Tables6^8^).

#### Comparison Between Hierarchical and Flat Classification

Using identical backbones, preprocessing steps, and data splits, we compared the hierarchical framework with the flat 3-class baseline.

On the internal test set, the hierarchical model achieved a higher balanced accuracy (hierarchical vs flat: 81.0% vs 77.1%), primarily driven by improved sensitivity for tendinopathy/partial-thickness tears (68.1% vs 57.4%) while maintaining comparable sensitivity for full-thickness tears (94.1% vs 95.1%).

On the independent external cohort, the hierarchical model again improved balanced accuracy (hierarchical vs flat: 68.1% vs 64.5%) and substantially increased sensitivity for tendinopathy/partial-thickness tears (45.5% vs 18.2%). This improvement was accompanied by an increase in false-positive predictions among intact cases, resulting in a modest reduction in overall accuracy (76.4% vs 79.8%).

These findings demonstrate an explicit sensitivity-specificity tradeoff as follows: by prioritizing the detection of full-thickness tears and subsequently resolving more subtle lesions, the hierarchical strategy reduces the underdiagnosis of partial-thickness pathology, which is clinically desirable, at the cost of increased false-positive predictions. Full performance metrics and confusion matrices for both models are provided in Tables6  7.

The detailed per-class precision, sensitivity, and *F*_1_-score for each model are provided in Table 9. Notably, the hierarchical framework consistently improved sensitivity for the tendinopathy/partial-thickness tear class compared with the flat baseline, particularly on the external cohort.

**Table 9.** Per-class performance of hierarchical and flat 3-class models on the internal and external test sets.

Dataset, model, and class		Precision	Sensitivity	*F*_1_-score
Internal test set (n=239)				
Hierarchical				
Intact tendons		0.900	0.811	0.853
Tendinopathy/partial-thickness tears		0.600	0.681	0.638
Full-thickness tears		0.914	0.941	0.928
Flat				
Intact tendons		0.877	0.789	0.831
Tendinopathy/partial-thickness tears		0.574	0.574	0.574
Full-thickness tears		0.863	0.951	0.905
External test set (n=89)				
Hierarchical				
Intact tendons		0.821	0.657	0.730
Tendinopathy/partial-thickness tears		0.385	0.455	0.417
Full-thickness tears		0.833	0.930	0.879
Flat				
Intact tendons		0.844	0.771	0.806
Tendinopathy/partial-thickness tears		0.400	0.182	0.250
Full-thickness tears		0.808	0.977	0.884

#### Leakage/Error-Propagation Analysis

Because hierarchical classification frameworks may be affected by error propagation, we conducted a leakage analysis to explicitly quantify misclassification attributable to each component model (model F and model ITP). The results are summarized in Table 10.

**Table 10.** Leakage analysis.

Dataset, model, and error type		Value, n
Internal test set (n=239)		
Model F^a^		
Full-thickness tears misclassified as non–full-thickness cases		6
Non–full-thickness cases misclassified as full-thickness tears		9
Model ITP^b^		
Tendinopathy/partial-thickness tears misclassified as intact tendons		7
Intact tendons misclassified as tendinopathy/partial-thickness tears		16
External test set (n=89)		
Model F		
Full-thickness tears misclassified as non–full-thickness cases		3
Non–full-thickness cases misclassified as full-thickness tears		8
Model ITP		
Tendinopathy/partial-thickness tears misclassified as intact tendons		4
Intact tendons misclassified as tendinopathy/partial-thickness tears		6

a Model F: classifier for differentiating full-thickness tears from non–full-thickness cases (intact tendons or tendinopathy/partial-thickness tears).

b Model ITP: classifier for distinguishing intact tendons from tendinopathy/partial-thickness tears.

##### Internal Test Set

In the hierarchical framework, model F (full-thickness vs non–full-thickness) misclassified 6 full-thickness tears as non–full-thickness cases (1 intact tendon and 5 tendinopathy/partial-thickness tear cases) and 9 non–full-thickness cases as full-thickness tears (1 intact tendon and 8 tendinopathy/partial-thickness tear cases), resulting in 15 irrecoverable routing errors. These cases bypassed subsequent classification by model ITP.

Among cases correctly routed as non–full-thickness cases, model ITP (tendinopathy/partial-thickness tears vs intact tendons) produced 23 errors, including 7 misclassifications of tendinopathy/partial-thickness tears as intact tendons and 16 misclassifications of intact tendons as tendinopathy/partial-thickness tears. These errors primarily reflect intrinsic visual overlap between intact tendons and subtle degenerative pathology on MRI.

By comparison, the flat 3-class model misclassified 13 tendinopathy/partial-thickness tear cases as full-thickness tears and 15 tendinopathy/partial-thickness tear cases as intact tendons, yielding a tendinopathy/partial-thickness tear sensitivity of 57.4%, which was lower than the value of 68.1% achieved using the hierarchical framework. Full-thickness sensitivity was comparable between models (hierarchical: 94.1%; flat: 95.1%).

##### External Test Set

In the external cohort, model F misclassified 3 full-thickness tears as non–full-thickness cases and 8 non–full-thickness cases as full-thickness tears, resulting in 11 irrecoverable routing errors. Model ITP had 10 additional errors, including 4 misclassifications of tendinopathy/partial-thickness tears as intact tendons and 6 misclassifications of intact tendons as tendinopathy/partial-thickness tears.

In contrast, the flat 3-class classifier demonstrated substantially reduced sensitivity for tendinopathy/partial-thickness tears, correctly identifying only 2 of 11 cases (sensitivity=18.2%), while the hierarchical model identified 5 of 11 cases (sensitivity=45.5%). This improvement in the most diagnostically challenging class was accompanied by an increase in false-positive tear predictions among intact cases (hierarchical: 6 vs flat: 2), resulting in a modest reduction in overall accuracy (hierarchical vs flat: 76.4% vs 79.8%) but a higher balanced accuracy (68.1% vs 64.5%).

## Discussion

### Principal Findings

This study describes a hierarchical deep learning framework for classifying supraspinatus tendon pathologies on coronal T2-weighted shoulder MRI. By separating the diagnostic task into 2 clinically guided stages—first detecting full-thickness tears and then distinguishing intact tendons from partial-thickness tears or tendinopathy—the model aligns with real-world diagnostic workflow patterns. The hierarchical design achieved high sensitivity for full-thickness tears and showed consistent performance on an external dataset, supporting its generalizability. Compared with a conventional flat model, the hierarchical approach was associated with numerically improved performance in selected metrics; however, the 95% bootstrapped CIs overlapped, suggesting that these differences should be interpreted with caution. Additionally, integration of Score-CAM enhanced model interpretability by highlighting clinically relevant features that informed model predictions. These results suggest that the AI-based framework described herein may support radiologists and orthopedic surgeons in improving diagnostic efficiency, consistency, and confidence in musculoskeletal imaging.

Recent advances in deep learning have correspondingly advanced the automated analysis of shoulder MRI for diagnosing rotator cuff tears. Several notable studies have explored various model architectures and approaches. Lin et al [24] developed an ensemble model using 4 parallel 3D ResNet-50 networks to classify rotator cuff tears as no tear, partial-thickness tear, or full-thickness tear. Guo et al [25] introduced a 2D CNN model based on the Xception architecture for the automated diagnosis of supraspinatus tears. Lee et al [26] used a YOLO v8–based model to detect rotator cuff tears across axial, coronal, and sagittal planes. Esfandiari et al [27] proposed a custom 2D CNN for binary classification of rotator cuff tears versus healthy cases. Yao et al [28] developed a 3-stage deep learning pipeline incorporating slice selection (ResNet), segmentation (U-Net), and a multi-input CNN classifier for supraspinatus tear detection.

While these prior studies demonstrated promising results, most were limited by single-center datasets and variable sample sizes—ranging from 100 to 11,000 MRI examinations—which may constrain generalizability and clinical applicability. In addition, prior studies differed substantially in class definitions, input configurations (eg, single-plane vs multisequence MRI), and imaging vendor characteristics, further limiting direct comparison across studies and hindering the assessment of real-world performance.

Recent work has demonstrated the feasibility of code-free deep learning (CFDL) platforms for musculoskeletal MRI analysis, including the detection and classification of supraspinatus tendon pathologies on shoulder MRI, enabling rapid model development without manual coding [29]. Similar advantages of CFDL approaches, such as accessibility and ease of use, have also been reported in other radiologic applications, including chest radiograph classification, detection, and segmentation [30].

However, code-free frameworks generally provide limited flexibility in task-specific preprocessing, explicit volumetric modeling, and structured decision strategies, which are critical for hypothesis-driven evaluation and clinical interpretability. In this study, a coded deep learning framework was therefore adopted to enable explicit 3D preprocessing, hierarchical task decomposition, and transparent analysis of sensitivity-specificity tradeoffs and error propagation. This design aligns with the clinical diagnostic workflow of rotator cuff evaluation and supports interpretable, clinically grounded model assessment. We did not adopt 2D slice-level aggregation approaches, as rotator cuff tear evaluation inherently requires the assessment of 3D spatial continuity across MRI slices. Clinically relevant features, such as tear extent, morphology, and tendon retraction, are distributed across multiple slices and cannot be reliably captured using isolated 2D representations, which may lead to suboptimal diagnostic performance.

Building on earlier work, this study has presented a hierarchical deep learning framework trained on 1192 shoulder MRI scans from a dataset that was predominantly derived from a single institution but also contained images collected across 65 institutions and acquired using 29 different scanner models. This dataset reflects some degree of real-world clinical variability and provides preliminary evidence regarding the robustness and generalizability of the model. Additionally, the new model integrates Score-CAM to provide intuitive, heatmap-based visualizations of the regions contributing to each prediction, supporting interpretability and offering clinical utility by assisting radiologists in verifying the model’s diagnostic rationale.

Although the classification task nominally involves only 3 diagnostic categories, the challenge lies not in the number of classes but in the substantial visual overlap between adjacent categories, particularly among intact tendons, partial-thickness tears, and early tendinopathy. In our flat 3-class baseline, this intrinsic ambiguity resulted in consistently low sensitivity for tendinopathy/partial-thickness tears, especially on the external cohort, despite using the same backbone architecture, preprocessing pipeline, and training configuration as in the hierarchical framework.

To address this limitation, we adopted a hierarchical classification strategy composed of 2 sequential binary decision steps, motivated by task decomposition rather than increased architectural complexity. In this framework, model F first identifies full-thickness tears and excludes them from further consideration, and then, model ITP classifies the remaining cases as intact or tendinopathy/partial-thickness tears. By explicitly separating a clinically high-priority decision from the assessment of more subtle pathology, the hierarchical design simplifies individual decision boundaries and closely mirrors diagnostic reasoning in routine clinical practice.

Quantitatively, the hierarchical model demonstrated numerically higher balanced performance compared with the flat 3-class baseline across both internal and external cohorts. Balanced accuracy increased from 77.1% to 81.0% on the internal test set and from 64.5% to 68.1% on the independent external cohort. However, the 95% bootstrapped CIs overlapped; therefore, these numerical differences should be interpreted cautiously and may not represent statistically significant differences. These differences were primarily driven by higher sensitivity for tendinopathy/partial-thickness tears—the most diagnostically challenging category—particularly in the external cohort (hierarchical vs flat: sensitivity 45.5% vs 18.2%). This represents a trend toward improved detection in this class, although the difference may not be statistically significant. This increase in sensitivity was accompanied by more false-positive predictions among intact cases, reflecting a sensitivity-specificity tradeoff. This tradeoff was explicitly characterized through per-class metrics and confusion matrices.

As expected, hierarchical models are susceptible to error propagation, whereby misclassification at an earlier decision stage cannot be corrected downstream. Our leakage analysis suggests that a substantial proportion of residual errors arose from clinically recognized ambiguity between intact tendons and tendinopathy/partial-thickness tears, rather than widespread routing failures in model F. Compared with the flat classifier, the hierarchical framework was associated with higher sensitivity for subtle tendon pathology, accompanied by reduced specificity for intact cases. This reflects a sensitivity-specificity tradeoff that may be clinically relevant; however, given the overlapping 95% bootstrapped CIs, these differences did not reach statistical significance. These findings should therefore be interpreted as a trend rather than a definitive improvement.

Finally, the 2-stage hierarchical architecture, together with the preliminary left-right shoulder classification used to ensure anatomical consistency of inputs, may enhance both interpretability and clinical plausibility. In addition to architectural design, several preprocessing strategies informed by imaging and clinical domain knowledge were incorporated and may have contributed to model performance.

The hierarchical framework was associated with numerically improved selected classification metrics and enhanced interpretability—an important factor for clinical integration. However, given the overlapping 95% bootstrapped CIs, these performance differences did not reach statistical significance. These findings suggest that structuring AI models in alignment with established diagnostic workflows may be beneficial, particularly in scenarios where certain pathology categories (eg, full-thickness tears) are visually distinct and diagnostically prioritized. Nevertheless, these observations should be interpreted as indicative of a trend, and further validation with larger cohorts and formal statistical testing is warranted.

The integration of Score-CAM further contributed to model transparency by generating interpretable heatmaps. Notably, attention maps from the left-right classifier and model F closely corresponded to anatomical regions of interest typically emphasized by experienced clinicians, suggesting that these models learned clinically relevant features. However, model ITP occasionally attended to regions inconsistent with the surgical focus, indicating a need for further refinement. Improving the model’s sensitivity to subtle findings, such as tendinopathy and partial-thickness tears, will likely require a larger, well-annotated dataset with richer representation of these pathologies. Notably, the evaluation of Score-CAM in this study was qualitative and based on representative examples rather than a systematic or quantitative assessment; therefore, these visualizations should be interpreted as supporting interpretability rather than providing definitive evidence of accurate anatomical localization.

### Strengths and Limitations

This study has several strengths. First, although the internal cohort was predominantly derived from a single institution, it also included MRI data from multiple external sources, providing an opportunity to preliminarily assess model performance across heterogeneous imaging settings. Second, the hierarchical classification strategy mirrored real-world diagnostic reasoning, contributing to numerically improved selected performance metrics and clinical interpretability.

This study also has several limitations. First, although volumetric slice standardization and manual quality control were implemented to ensure supraspinatus tendon coverage, it remains possible that peripheral or atypically positioned tear extensions were not fully captured within the standardized 16-slice volume. This design choice balanced anatomical coverage and computational feasibility. Future work may explore adaptive slice selection or attention-based volumetric models. Second, the external validation cohort—while useful for assessing generalizability—contained only 11 cases of tendinopathy or partial-thickness tears. This limited subgroup sample size introduced statistical fragility and wide CIs, restricting the strength of conclusions regarding external generalizability for this category. Sensitivity estimates for tendinopathy/partial-thickness tears should therefore be interpreted with caution, and larger external cohorts will be required to verify performance for this subgroup. Third, potential label bias may exist, as full-thickness tears were confirmed using surgical or arthroscopic findings, while partial-thickness tears and tendinopathy were labeled based on imaging consensus. This difference in reference standards may introduce classification bias. Accordingly, the model’s performance for partial-thickness tears and tendinopathy reflects agreement with expert interpretation rather than true diagnostic accuracy against a surgical gold standard, which may limit the achievable performance for these classes. Fourth, the hierarchical framework may be susceptible to cumulative error propagation, in which misclassifications at early stages influence downstream outputs. This tradeoff reflects an intentional prioritization of sensitivity for partial-thickness tears over global accuracy but may reduce specificity for certain subgroups. Fifth, the model was trained exclusively on coronal T2-weighted sequences, whereas ground truth labels were established based on full multiplanar and multisequence clinical MRI review. This modality gap may limit the recognition of subtle partial-thickness tears, which often benefit from sagittal or proton-density fat-suppressed views. Future work incorporating multisequence or multiplane inputs may enhance diagnostic performance. Sixth, tendinopathy and partial-thickness tears were grouped into a single category to reflect overlapping early management in routine clinical practice. However, this grouping reduces granularity for surgical planning, particularly around thresholds for operative referral versus conservative management. Seventh, patients with moderate-to-severe glenohumeral osteoarthritis (KL grades 2‐4) were excluded, which may limit applicability to older individuals at the highest risk for degenerative rotator cuff pathology and introduce a form of selection bias. Prospective validation in more heterogeneous clinical populations is warranted. Eighth, with respect to the left-right classification task, although all MRI volumes were visually inspected to confirm the absence of burned-in laterality markers or textual overlays, the possibility that subtle nonanatomical cues contributed to the classification cannot be completely excluded. Ninth, the assessment of Score-CAM was qualitative and lacked formal radiologist-based or quantitative validation, and thus, the extent to which the highlighted regions correspond to true anatomical focus remains uncertain. Tenth, most cases originated from a single hospital, TVGH, which may introduce center-specific imaging bias and limit the model’s generalizability to other institutions. Finally, alternative model architectures, including 2D slice-level ensembles or transformer-based volumetric models, were not explored in order to isolate the effect of hierarchical diagnostic task structuring. Future studies may investigate hybrid approaches to further align model behavior with clinical decision-making pathways.

### Conclusions

In this study, we developed a hierarchical deep learning framework to classify supraspinatus tendon pathologies on shoulder MRI. The framework decomposed the diagnostic task into two binary stages—first distinguishing full-thickness tears and then differentiating intact tendons from tendinopathy or partial-thickness tears—reflecting clinical diagnostic reasoning. Across internal and external evaluations, the hierarchical approach showed numerically improved recognition of tendinopathy and partial-thickness tears relative to a conventional flat 3-class model while maintaining high sensitivity for full-thickness tears. These findings suggest that clinically motivated task structuring may support more reliable supraspinatus pathology classification and may align with radiological interpretation workflows, although further validation with larger cohorts and formal statistical testing is warranted.

## Supplementary material

10.2196/84804 Multimedia Appendix 1 The slice thickness, slice spacing, and XY spacing of the internal cohort (case-level) and image-level dataset composition.

10.2196/84804 Multimedia Appendix 2 Heatmap of the models and training processes for the hierarchical models.

## Abbreviations

AI: artificial intelligence
AUC: area under the curve
CFDL: code-free deep learning
CNN: convolutional neural network
KL: Kellgren-Lawrence
MRI: magnetic resonance imaging
ROC: receiver operating characteristic
Score-CAM: Score-weighted Class Activation Mapping
TVGH: Taipei Veterans General Hospital
