# Supplementary material for: AI-Assisted Detection of Supraspinatus Tendon Pathologies Using a Hierarchical Deep Learning Model to Improve Clinical Applicability: Development and Evaluation Study
Source: JMIR Med Inform. 2026 Jul 8;14:e84804. doi: 10.2196/84804 (PMC13345813; doi:10.2196/84804)
Supplement: Multimedia Appendix 1 [file medinform-v14-e84804-s001.docx]

**Table S1. The slice thickness, slice spacing, and X Y spacing of Internal Cohort (case-level)**

1. Slice thickness (mm)

| **Slice thickness** | **Training (n=764)** | **Validation (n=189)** | **Internal test (n=239)** | **Internal Total (n=1192)** | **External (n=89)** |
| --- | --- | --- | --- | --- | --- |
| **2.0** | 4 (0.5) | 1 (0.5) | 1 (0.4) | 6 (0.5) | 0 (0.0) |
| **3.0** | 609 (79.7) | 157 (83.1) | 192 (80.3) | 958 (80.4) | 79 (88.8) |
| **3.5** | 15 (2.0) | 5 (2.6) | 5 (2.1) | 25 (2.1) | 2 (2.2) |
| **4.0** | 135 (17.7) | 26 (13.8) | 41 (17.2) | 202 (16.9) | 8 (9.0) |
| **4.5** | 1 (0.1) | 0 (0.0) | 0 (0.0) | 1 (0.1) | 0 (0.0) |

1. Slice spacing (mm)

| **Slice spacing** | **Training (n=764)** | **Validation (n=189)** | **Internal test (n=239)** | **Internal Total (n=1192)** | **External (n=89)** |
| --- | --- | --- | --- | --- | --- |
| **2.0** | 2 (0.3) | 0 (0.0) | 0 (0.0) | 2 (0.2) | 0 (0.0) |
| **3.0** | 86 (11.3) | 23 (12.2) | 23 (9.6) | 132 (11.1) | 6 (6.7) |
| **3.3** | 58 (7.6) | 17 (9.0) | 29 (12.1) | 104 (8.7) | 59 (66.3) |
| **3.48** | 20 (2.6) | 4 (2.1) | 2 (0.8) | 26 (2.2) | 1 (1.1) |
| **3.5** | 371 (48.6) | 90 (47.6) | 113 (47.3) | 574 (48.2) | 8 (9) |
| **3.6** | 10 (1.3) | 2 (1.1) | 1 (0.4) | 13 (1.1) | 2 (2.2) |
| **3.7** | 0 (0.0) | 0 (0.0) | 0 (0.0) | 0 (0.0) | 3 (3.4) |
| **3.85** | 1 (0.1) | 1 (0.5) | 0 (0.0) | 2 (0.2) | 0 (0.0) |
| **3.9** | 12 (1.6) | 3 (1.6) | 5 (2.1) | 20 (1.7) | 0 (0.0) |
| **3.99** | 0 (0.0) | 0 (0.0) | 1 (0.4) | 1 (0.1) | 0 (0.0) |
| **4.0** | 175 (22.9) | 44 (23.3) | 56 (23.4) | 275 (23.1) | 2 (2.2) |
| **4.2** | 1 (0.1) | 0 (0.0) | 0 (0.0) | 1 (0.1) | 0 (0.0) |
| **4.4** | 14 (1.8) | 2 (1.1) | 5 (2.1) | 21 (1.8) | 7 (7.7) |
| **4.5** | 10 (1.3) | 2 (1.1) | 4 (1.7) | 16 (1.3) | 1 (1.1) |
| **4.8** | 1 (0.1) | 0 (0.0) | 0 (0.0) | 1 (0.1) | 0 (0.0) |
| **5.0** | 3 (0.4) | 1 (0.5) | 0 (0.0) | 4 (0.3) | 0 (0.0) |

1. In-plane pixel spacing (X, Y; mm, grouped)

| **Pixel spacing range (mm)** | **Training (n=764)** | **Validation (n=189)** | **Internal test (n=239)** | **Internal Total (n=1192)** | **External (n=89)** |
| --- | --- | --- | --- | --- | --- |
| **0.1–0.19** | 0 (0.0) | 1 (0.5) | 0 (0.0) | 1 (0.1) | 0 (0.0) |
| **0.2–0.29** | 36 (4.7) | 15 (7.9) | 18 (7.5) | 69 (5.8) | 17 (19.1) |
| **0.3–0.39** | 644 (84.3) | 158 (83.6) | 196 (82.0) | 998 (83.7) | 58 (65.2) |
| **0.4–0.49** | 59 (7.7) | 15 (7.9) | 15 (6.3) | 89 (7.5) | 14 (15.7) |
| **0.5–0.59** | 14 (1.8) | 0 (0.0) | 9 (3.8) | 23 (1.9) | 0 (0.0) |
| **0.6–0.69** | 9 (1.2) | 0 (0.0) | 0 (0.0) | 9 (0.8) | 0 (0.0) |
| **0.7–0.79** | 1 (0.1) | 0 (0.0) | 1 (0.4) | 2 (0.2) | 0 (0.0) |
| **0.8–0.89** | 1 (0.1) | 0 (0.0) | 0 (0.0) | 1 (0.1) | 0 (0.0) |

- n (%): n denotes the number of cases, and percentages are calculated within each dataset. Percentages are not shown for the total column.
- Pixel spacing values were grouped by the first decimal digit for readability.

**Table S2. Image-level dataset composition**

(a) Overall image distribution

| **Dataset** | **Number of images** |
| --- | --- |
| Training set | 14,431 |
| Validation set | 3,499 |
| Internal test set | 4,599 |
| **Internal total** | **22,529** |
| External set | 2,219 |

- Image counts correspond to raw DICOM slices prior to 16-slice volumetric standardization.

(b) Image-level distribution by shoulder side

| **Shoulder side** | **Training (n=14,431)** | **Validation (n=3,499)** | **Internal test (n=4,599)** | **External (n=2,219)** |
| --- | --- | --- | --- | --- |
| Right | 9,545 (66.1) | 2,308 (66.0) | 3,071 (66.8) | 1,393 |
| Left | 4,886 (33.9) | 1,191 (34.0) | 1,528 (33.2) | 826 |

(c) Image-level distribution by tear type

| **Tear type** | **Training (n=14,431)** | **Validation (n=3,499)** | **Internal test (n=4,599)** | **External (n=2,219)** |
| --- | --- | --- | --- | --- |
| Intact | 5,235 (36.3) | 1,288 (36.8) | 1,663 (36.2) | 879 |
| Partial-thickness tear / Tendinopathy | 2,853 (19.8) | 715 (20.4) | 891 (19.4) | 273 |
| Full-thickness tear | 6,343 (43.9) | 1,496 (42.8) | 2,045 (44.5) | 1,067 |

- n (%): n denotes the number of cases, and percentages are calculated within each dataset.
- Image-level statistics are provided for completeness; all analyses in the main manuscript were conducted at the case level.
