# Supplementary material for: AI-Assisted Detection of Supraspinatus Tendon Pathologies Using a Hierarchical Deep Learning Model to Improve Clinical Applicability: Development and Evaluation Study
Source: JMIR Med Inform. 2026 Jul 8;14:e84804. doi: 10.2196/84804 (PMC13345813; doi:10.2196/84804)
Supplement: Multimedia Appendix 2 [file medinform-v14-e84804-s002.docx]

**
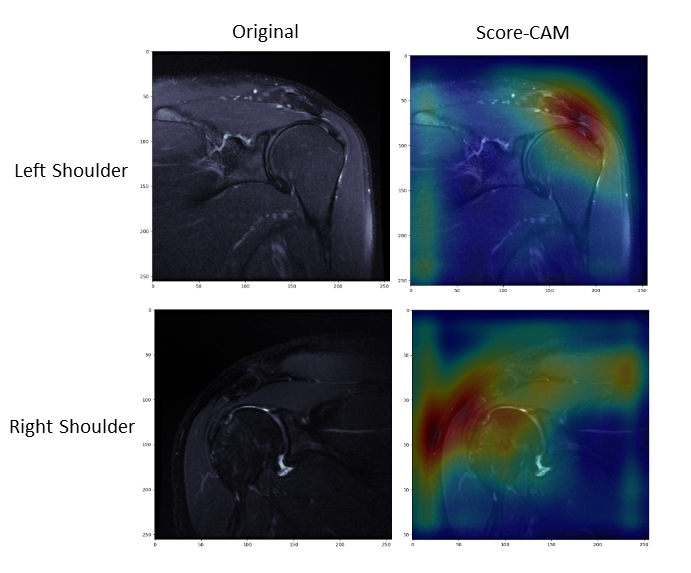
**

**Figure S1.** Heatmap of the left-right shoulder model.

**
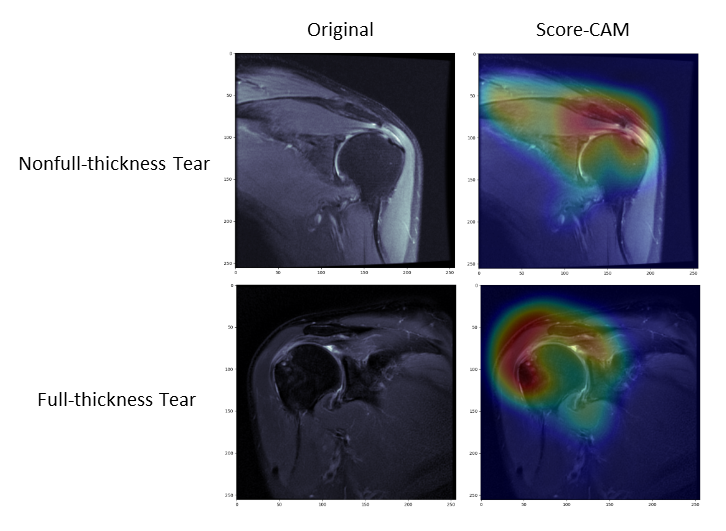
**

**Figure S2.** Heatmap of Model F.

**
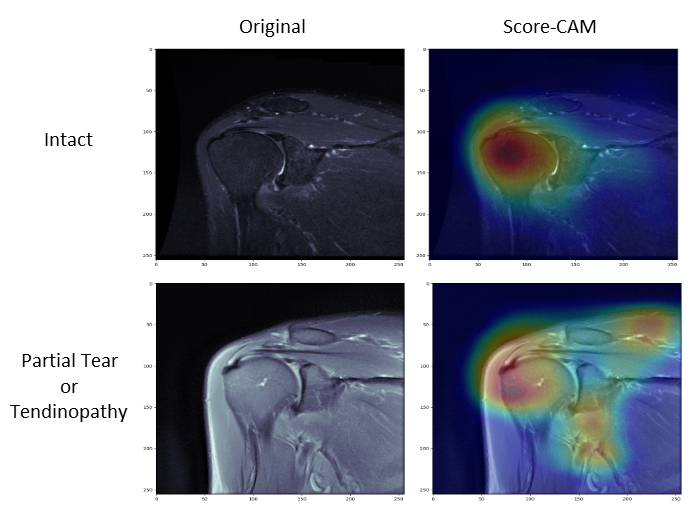
**

**Figure S3.** Heatmap of Model ITP.

**
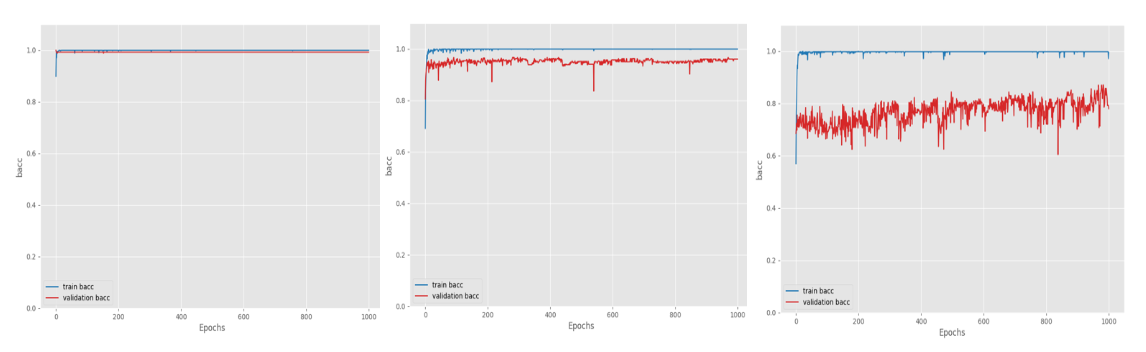
**

**Figure S4.** Training processes for the hierarchical models: left vs. right shoulder (left), full-thickness vs. non–full-thickness tears (middle), and intact vs. tendinopathy/partial-thickness tears (right).
